# Perspectives of choice and control in daily life for people following brain injury: A qualitative systematic review and meta‐synthesis

**DOI:** 10.1111/hex.13636

**Published:** 2022-10-31

**Authors:** Carolyn M. Murray, Scott Weeks, Gisela van Kessel, Michelle Guerin, Emma Watkins, Shylie Mackintosh, Caroline Fryer, Susan Hillier, Mandy Stanley

**Affiliations:** ^1^ Allied Health and Human Performance Academic Unit University of South Australia Adelaide South Australia Australia; ^2^ University of South Australia Adelaide South Australia Australia; ^3^ Neuromoves Adelaide South Australia Australia; ^4^ School of Medical and Health Sciences Edith Cowan University Joondalup Western Australia Australia

**Keywords:** behavioural sciences, brain injuries, traumatic, emotions, health services, rehabilitation, social sciences, stroke

## Abstract

**Background and Objective:**

Acquired brain injury (ABI) can result in considerable life changes. Having choice and control over daily life is valued by people following ABI. This meta‐synthesis will analyse and integrate international research exploring perspectives of choice and control in daily life following ABI.

**Methods:**

Databases were searched from 1980 to 13 January 2022 for eligible qualitative studies. After duplicates were removed, 22,768 studies were screened by title and abstract, and 241 studies received full‐text assessment with 56 studies included after pearling. Study characteristics and findings were extracted that related to personal perspectives on choice and control by people with an ABI (including author interpretation and quotes). Data from each study were coded and then segments of coded data across the studies were compared to create multiple broad categories.

**Findings:**

Findings were then reduced from categories into 3 overarching themes with 12 subthemes. These themes were: (1) feeling like a second‐class citizen; (2) reordering life and (3) choosing a path. Participants with an ABI tussled between their feelings of loss following brain injury and their thinking about how they start to regain control and become agents of their own choices. The themes describe their sense of self, their changed self and their empowered self in relation to ‘choice and control’.

**Conclusions:**

Re‐engaging with choice and control after ABI is dynamic and can be challenging. Health professionals and supporters need to facilitate a gradual and negotiated return to agency for people following ABI. A sensitive and person‐centred approach is needed that considers the readiness of the person with ABI to reclaim choice and control at each stage of their recovery. Clear service or process indicators that are built on lived experience research are needed to facilitate changes in service delivery that are collaborative and inclusive.

**Patient or Public Contribution:**

This review included the voices of 765 people living with ABI and was conducted by a diverse team of allied health professionals with practice knowledge and research experience with people following ABI. Twenty‐nine of the 56 included studies had participants contributing to their design or analysis.

## 1 INTRODUCTION

Acquired brain injury (ABI) is an overarching term for brain damage acquired from stroke, infections, toxins, tumours, hypoxia or traumatic brain injury (TBI).^1^ The most common categories within ABI are TBI and stroke. Sixty‐nine million individuals have been estimated to experience ABI each year, with the Southeast Asian and Western Pacific regions having the greatest overall incidence.^2^ ABI may result in cognitive, physical or emotional impairments and reduced independent functioning^3^ leading to long‐term and complex disability.^4^, ^5^ Limitations that result from ABI may continue for decades and relate to ongoing participation restrictions.^4^ ABI can impact a person's engagement^6^ including difficulties with performing activities of daily living, participating in a full round of social activities with friends and family and engaging in work or study and caring for family members commensurate with one's life stage.

In the first 3–6 months after returning home following ABI, there is a growing self‐awareness of new capacities and the need for meaningful activities.^7^ This phase requires flexible support services that facilitate the transition to the community. The complexity and rigidity of service systems impact transition success and may amplify difficulties that the person with ABI and their caregivers experience during transition.^8^ People with ABI and their families have reported being excluded and restricted by authorities.^7^ Conversely, they have also reported feeling supported by people who listened to them and demonstrated a desire to understand rather than judge.^9^ Therapists need to be skilled in collaborative goal‐setting, weighing up risks and autonomy as a person‐centred interaction, using critical elements of control, empowerment, support and advocacy.^9^

Regaining control over everyday life has been linked with feeling well by people with moderate to severe ABI,^10^ suggesting choice and control are important. Globally, particularly in Western countries, consumer choice and control are attracting attention. Personalization of social care in the United Kingdom,^11^ consumer‐directed care in the United States of America, United Kingdom, Australia and New Zealand^12^ and the introduction of the National Disability Scheme in Australia^13^ have all shifted the focus of care systems to consumer choice and control. However, how effectively they support all consumers to have control of their choices in health care or life, in general, has been questioned.^13^, ^14^ These questions relate to consumers having the skills and knowledge to advocate for their needs in a bureaucratic environment,^13^, ^14^ leaving them vulnerable and reliant on the competence and skills of those providing the services.^15^ A 2019 systematic review for people after spinal cord injury concluded that exercising choice and control requires a complex interplay of systems and that protective and insensitive attitudes of the people around the person with SCI (health professionals, family, support people) can create barriers.^16^ A study exploring opportunities for choice and control in Swedish individualized home care for older people highlighted the importance of supportive relationships, and the interdependence between older people and their formal, as well as informal, support networks to have an effective choice of home care services.^17^

Factors facilitating choice and control may differ across health conditions and demographics. While a framework that focuses on understanding the preferences of people with cognitive disabilities^18^ can enable better experiences of shared decision‐making, there has been limited application to people with ABI. To our knowledge, no review has conducted a synthesis of the literature exploring the experiences of adults with ABI regarding choice and control. Therefore, this review will address the question: *What are the views of people living in the community with ABI on their ability to exercise choice and control in their daily life?*

## 2 METHODS

### 2.1 Study design

Meta‐synthesis using the meta‐ethnographic approach.^19^ Our review was registered with the International Prospective Register of Systematic Reviews, PROSPERO, in May 2016 (CRD42016038680). The protocol followed the Enhanced Transparency of Reporting the Synthesis of Quality Research (ENTREQ) reporting guide^20^ and the PRISMA for systematic reviews.^21^

### 2.2 Search strategy

Initially, 13 electronic databases were searched from 1980 to May 2016—Medline, EMBASE, Health‐Society, Humanities & Social Sciences Collections, PsychInfo, ProQuest (Social Science), Cochrane, SCOPUS, CINAHL, Academic Search Premier, Health Source (Nursing/Academic Edition), Psychology and Behavioural Sciences Collection and SAGE (Health Sciences). Database searches were rerun from 2016 up to 13 January 2022 with EMCARE replacing CINAHL and Social Science Premium Collection replacing Proquest. Academic Search Premier, Health Source (Nursing/Academic Edition), Psychology and Behavioural Sciences Collection and SAGE (Health Sciences) were not searched as these databases were no longer available.

Search terms included subject headings and free‐text words related to ‘choice’, ‘control’, ‘acquired brain injury’ and ‘personal perspectives’. The search terms were reviewed by an academic librarian and conducted initially in Medline and then adapted for use in each database. Search parameters were limited to human‐only and studies published after 1980 because this era marked the beginning of the deinstitutionalization of people living with ABI. The complete Medline search is outlined in Supporting Information: Appendix S1.

### 2.3 Study selection

Duplicate articles were removed using EndNote™X9 (www.endnote.com; Clarivate Analytics), then exported into Covidence™ (www.covidence.org; Veritas Health Innovation) and two reviewers independently screened titles and abstracts. Full‐text articles were then reviewed independently by two reviewers. Conflicts were resolved by a third independent reviewer. Reference lists of included studies were hand‐searched to source additional papers.

### 2.4 Inclusion and exclusion criteria

Qualitative studies were included. Mixed methods studies were included if the qualitative data was separate. Full inclusion and exclusion criteria are explained in Table 1. The definition of choice and control was taken from a similar systematic review with a different population: ‘choice was the opportunity to make a decision when two or more options were presented. Control was the ability to influence an action or course of events’ (Murray et al.^16^,p.5). Whilst the focus of the review was on community experiences, it became clear that hospital experiences of choice and control are relevant once people leave the hospital and these perspectives could not be separated. Therefore, when studies included community participants reflecting on their hospital/inpatient experience, these data were also extracted.

**Table 1:** Inclusion and exclusion criteria of papers

	Inclusion criteria	Exclusion criteria
Population	Participants over 18 years living with acquired brain injury Studies with participants younger than 18 were only included if data could be extracted separately for those over 18 years or over 75% of participants were over 18 years Studies with multiple populations (e.g., carers, health professionals and people living with ABI) were only included if the data could be extracted separately for those living with an ABI	Participants younger than 18 Participants who were carers or health professionals
Interest	Studies exploring choice and control following brain injury	insufficient detail to inform our understanding of the concepts of choice and control
Context	Living in the community undertaking their daily life Data with participants reflecting on their inpatient experience were included Papers including participants with mixed living circumstances (i.e., community, residential and hospital) were included if the data from community participants could be extracted separately or over 75% of the sample were community dwelling	Living in residential care or in hospital/inpatient care at the time of interview

Abbreviation: ABI, acquired brain injury.

### 2.5 Critical appraisal of selected papers

The McMaster Critical Appraisal Tool^22^ for qualitative research was conducted in duplicate on all studies (C. M. M., G. v. K., C. F., E. W.).

### 2.6 Data extraction and synthesis

Data were extracted into a customized spreadsheet independently by two reviewers. We followed the process outlined by Noblit and Hare^19^ for analysing and synthesizing qualitative literature within the healthcare context. The first‐order analysis involved two reviewers independently reading the full text of included studies and identifying findings related to ‘choice and control’ in everyday life (M. G., E. W., C. M. M., G. v. K.). These findings (including author interpretation and participant quotes) were extracted, and each segment of data coded. The second‐order analysis had two steps, the first step involved three authors meeting to sort the coded data into groups to recognize categories across the studies^23^ (C. M. M., E. W., S. W.). The second step involved the wider research team discussing the findings within the context of the original papers, and using a narrative approach to further refine the lines of argument.^23^ Third‐order analysis involved further reduction and abstraction of the categories to form higher‐order themes. At all stages, there were regular meetings with members of the research team to discuss data interpretation and conceptual development.

### 2.7 Rigour

To ensure the rigour of the review process, all papers were screened in duplicate thus minimizing the risk of bias in inclusion. All members of the research team have a background as allied health clinicians in ABI rehabilitation and recovery. Having multiple researchers involved in the research process from different disciplines and levels of experience ensured robust discussion at all decision points, particularly during the final stage of analysis. An audit trail of analytic decisions was kept through the analysis process (January 2021 to June 2022). Pearling of the included studies resulted in the inclusion of a further four papers. Data from these new studies were consistent with existing findings, therefore reaching saturation.^24^

## 3 FINDINGS

### 3.1 Search outcomes

**Figure 1:**
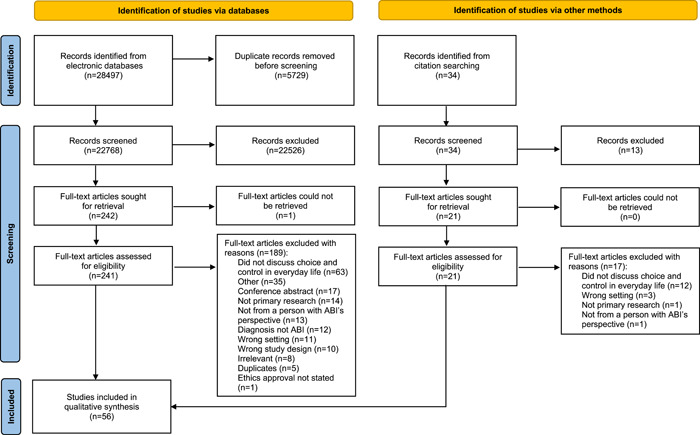
PRISMA flow diagram

There were 22,768 studies screened by title and abstract with 241 studies retrieved for full‐text review. After a full‐text review and pearling reference lists of included studies, 56 studies met the eligibility criteria. The reasons for exclusion are specified in Figure 1. One eligible study was in French and was translated for inclusion.^25^ One study in German could not be located and was excluded at the title and abstract.

### 3.2 Study characteristics

Fifty‐six studies were included from 13 countries, including Australia (*n* = 13), United Kingdom (*n* = 10), Canada (*n* = 10), United States (*n* = 6), Norway (*n* = 5), Sweden (*n* = 5) and 1 paper from Ireland, France, Denmark, Uganda, Portugal, the Netherlands and New Zealand. Twenty‐nine studies (52%) had public and participant involvement either through co‐design/consultation, piloting of interview guides or member checking. A summary of the study aims and participant numbers for each paper is provided in Table 2 (more detail in Supporting Information: Appendix S2). There were 765 people with ABI contributing to the findings with further details of participants provided in Table 3. The majority (54.5%) were male and within 2 years since their ABI (39.2%). Of the 56 papers, 20 deliberately included people with communication difficulties, 23 excluded people with communication difficulties and 13 did not report on participant communication.

**Table 2:** Breakdown of study aim and participant gender and age^a^

References			Author (year) country	Number and gender	Age of participants	Aim
[26]			Allen (2021) Ireland	14^b^; 8m, 6f	Mean age 47	Experience of living with brain injury according to personal growth
[27]			Anderson (2013) Canada	9; 6m, 3f	Age range 53–64	Resources that enhance activity participation after stroke
[28]			Arntzen (2014) Norway	9; 6m, 3f	Age range 39–72	Long‐term negotiations and recovery trajectory
[29]			Berg (2017) Norway	15; 7m, 8f	Age range 43–74; median 61	Participation in goal setting with aphasia
[30]			Boger (2015) UK	28; 11m, 17f	Mean age 65.67	Factors facilitating or hindering stroke self‐management
[31]			Burton (2000) UK	6; 4m, 2f	Mean age 67	For people to describe their own recovery.
[32]			Carulli (2018) USA	12; 8m, 4f	Age range 18–36; mean 24.8	Student social engagement
[33]			Conneeley^c^ (2002) UK	18; 13m, 5f	Mean age 35^h^	Explore social integration issues following rehabilitation
[24]			Coneeley^c^ 2003) UK	18; 13m, 5f	Mean age 35^h^	Quality of life after rehab
[34]			Conneeley^c^ (2012) UK	18; 13m, 5f	Mean age 35^h^	Explore the transition from hospital to home
[23]			Dumont (2007) France	53; 37m, 16f	Mean age 37.5	Adaptation process and coping strategies
[35]			Finch (2020) Australia	17; 12m, 5f	Mean age 68.29	Experience following minor stroke
[36]			Fraas (2009) UK	31; 21m, 10f	Mean age 44	Factors for successful recovery and lifestyle
[37]			Gallagher (2011) Canada	9; 5m, 1f	Age range 42–82	Process of emotional recovery
[38]			Gould (2019) Australia	11m^d^ (4 eligible^e^)	Age range 29–56; mean 44.2	Experience of behaviours of concern
[39]			Graff (2020) Denmark	22; 8m, 14f	Age range 24–60	Barriers and facilitators to returning to work
[25]			Green (2009) Canada	26m	Age range 48–82; mean 64	Impact of quality of life
[40]			Häggström (2008) Sweden	11; 5m, 6f	Age range 38–62; mean 55	Experience of participation in daily life
[41]			Hammond (2021) USA	57^d^ (54 eligible^b^); 41m; 16f	Mean age at injury 41.2	Political participation after brain injury
[42]			Harrington (2015) Australia	10; 9m, 1f	Age range 20–50	Experiences of pathways, outcomes and choice
[43]			Harris Walker (2021) USA	20; unknown gender	Median age 52.5	Influences on recovery for younger adults
[44]			Herrmann (2019) USA	6; 2m, 4f	Age range 65–80	Experiences of hospitalization and recovery
[45]			Johansson (2016) Norway	8m	Age range 30–60	Daily activities and roles for returning to work
[46]			Jones (2008) UK	10; 6m, 4f	Age range 29–75; mean 61.8	Personal factors and resources to support recovery
[10]			Jumisko (2009) Sweden	8; 6m, 2f	Age range 29–53; median 41	The meaning of feeling well
[47]			Kamwesiga (2016) Uganda	11; 6m, 5f	Age range 25–75	Experience of using mobile phones
[48]			Kelly (2021) Australia	6 (4 eligible^e^) 3m; 1f	Age range 30–69	Rehabilitation for Aboriginal Australian people
[49]			Kessler (2009) Canada	12; 10m, 2f	Age range 44–74; mean 54	The process of change during recovery
[50]			King (2018) USA	22; 19m, 3f	Mean age 45	Factors that inform beliefs
[51]			Kitson (2013) UK	15; 6m, 9f	Age range mid 30s–mid 80s	Experience of fundamentals of care
[52]			Knox (2016)^f^ Australia	4; 3m, 1f	Age range 27–47	Understanding decision‐making
[53]			Knox (2017)^f^ Australia	7^e^; 4m. 3f	Age range 20–59	Exploring decision making and self‐concept
[54]			Koller (2016) Canada	6; 5m, 1f	Age range 37–51; mean 44.5	Experiences of financial management
[55]			Kubina (2013) Canada	6; 3m, 3f	Age range 40–68; mean 58	Reengagement in activities
[56]			Kusec (2020) Canada	21; 18m, 3f	Age range 32–64; mean 47.7	Engagement in community‐based programmes
[57]			Lawson (2008) Canada	1f	Age not specified	Personal narrative of rehabilitation
[58]			McCluskey (2007) Australia	14; 8m, 6f	Age range 19–56; mean 36.5.	The process of care management
[59]			Mealings (2021) Australia	9^g^; 8m, 1f	Age range 18–30	Student participation
[60]			Moss (2021) UK	20; 10m, 10f	Age range 25–85	Psychosocial adjustment with aphasia
[61]			Nalder (2013) Australia	16; 15m, 1f	Age range 18–55	Experiences of going home
[62]			Olofsson (2005) Sweden	9; 4m, 5f	Age range 64–83; mean 72	Reflections on hospital and going home experiences
[63]			Paniccia (2019) Canada	13; 5m, 8f	Age range 18–25^g^	Transition to work roles
[64]			Pereira (2020) Portugal	8; 6m, 2f	Age range 43–79; mean 66	Perspectives on adaptation over time
[65]			Price (2012) USA	1m	In his 70s	Narrative about resilient adaptation
[66]			Quinn (2014) UK	8; 7m, 1f	Age range 36–65	Experience of young couples after stroke
[67]			Ringsberg (2003) Sweden	15; 11m, 4f	Age range 59–85; mean 69	Perspectives of home rehabilitation
[68]			Satink (2016) the Netherlands	10; 4m, 6f	Age range 54–77	Self‐management through everyday activities
[69]			Sveen (2016) Norway	20; 8m, 12f	Age range 22–60; mean 40	Everyday occupations and return to work participation
[70]			Taule (2015) Norway	8; 4m, 4f	Age range 45–80	Experiences of home rehab
[71]			Timothy (2016) NZ	6^e^; 5m, 2f	Age range 66–89	Embodiment while transitioning to home
[72]			Tomkins (2013) Australia	50; 24m, 26f	Mean age 63.9	Satisfaction with health care
[73]			Turner (2009) Australia	20;15m, 5f	Age range 17–63^h^	Reengagement in meaningful occupation for youth
[74]			Vestling (2013) Sweden	12; 8m, 4f	Age range 43–61; mean 53	Thoughts and feelings about return to work
[75]			Walder (2017) Australia	6; 2m, 4f	Age range 34–76	Re‐establishing occupational identity
[76]			Wolfenden (2015) Australia	5f	Age range 28–44	Vulnerability of younger high functioning people
[77]			Wood (2010) Canada	10; 6m, 4f	Age range 31–79	Process of community reintegration

Abbreviations: f, female; m, male; NZ, New Zealand; TBI, traumatic brain injury; UK, United Kingdom; USA, United States of America.

^a^ Key: number of participants depends on reporting.

^b^ Living circumstance not given but presume more than 75% in the community.

^c^ Same research with different focus and participants only counted once.

^d^ Mean age indicates 75% of participants over 18—all TBI participant data extracted.

^e^ Demographic information includes people who were not interviewed.

^f^ Participants in residential care/supported accommodation/hospital not included.

^g^ May be the same study with different focus but not explained and unable to compare across studies due to different details given—all participants included in total.

^h^ Only participants over 18 reported and extracted.

**Table 3:** Participant details from included papers

	Participants (*n* = 765)
Age (years)	
18–39 (%)	131 (17)
40–65 (%)	187 (24.5)
65 plus (%)	79 (10.5)
Unknown age^a^ (%)	368 (48)
Gender	
Male (%)	417 (54.5)
Female (%)	221 (29)
Unknown gender (%)	127 (16.5)
Time since injury (years)	
0–2 (%)	300 (39.2)
3–5 (%)	48 (6.3)
6–10 (%)	48 (6.3)
11 plus (%)	47 (6.2)
Not reported for individuals^a^ (%)	322 (42)
Type of injury	
Stroke	418 (54.7)
Trauma	339 (44.3)
Other	8 (1)

^a^ Some papers reported ages and time since injury in means and ranges without giving individual participant details. Where ages were given with quotations or samples were small, it was possible to estimate the age range and time since the injury of participants. However, this was not always possible, particularly for larger samples with wide age ranges.

### 3.3 Critical appraisal findings

The papers clearly described relevant background literature and their study design was appropriate for the study purpose. Consistently, papers did not provide details about the bias and assumptions of the researchers and their relationship with participants, which affected overall confirmability. Similarly, the lack of information about the site where the data collection occurred, the demographics of participants and sampling limited the transferability of the research. Credibility was evident through having teams of researchers and using member checking. No papers were excluded based on quality; one paper was excluded as ethics approval was unable to be confirmed. There were five papers where ethics approval was explained but the process of informed consent was not. These papers were still included as consent was presumed based on having ethical approval. Refer to Supporting Information: File S3 for an overview of the critical appraisal findings.

### 3.4 Meta‐synthesis findings

The third‐order analysis confirmed three higher‐order themes, each with four subthemes. These were ‘feeling like a second‐class citizen’, ‘reordering life’ and ‘choosing a path’. The findings demonstrate how the participants tussle between their feelings of loss following ABI and their thinking about how to regain control and become agents of their own choices. These themes describe their sense of self, their changed self and their empowered self in relation to ‘choice and control’. There are various stages of readiness for acceptance with opportunities for choice and control being pivotal to their experience in changing agency and identity. Details of which papers contributed to the themes and subthemes can be found in Table 4. Each theme and subtheme are illustrated with quotes provided in Table 5.

**Table 4:** Papers contributing to subthemes

No.	Theme	Citations	Number of papers
1	Feeling like a second‐class citizen		
	Being depersonalized	^10^, ^23^, ^24^, ^25^, ^26^, ^27^, ^28^, ^29^, ^31^, ^32^, ^35^, ^37^, ^43^, ^44^, ^46^, ^49^, ^50^, ^51^, ^53^, ^57^, ^58^, ^60^, ^66^, ^67^, ^70^, ^71^, ^76^, ^77^	28
	Profound loss of self	^23^, ^24^, ^25^, ^26^, ^27^, ^28^, ^31^, ^32^, ^33^, ^34^, ^35^, ^37^, ^38^, ^40^, ^41^, ^48^, ^50^, ^51^, ^52^, ^53^, ^58^, ^59^, ^60^, ^61^, ^63^, ^66^, ^67^, ^69^, ^70^, ^72^, ^73^, ^74^, ^75^, ^76^	34
	Isolated and trapped	^24^, ^25^, ^26^, ^31^, ^33^, ^34^, ^35^, ^36^, ^37^, ^40^, ^41^, ^44^, ^45^, ^46^, ^47^, ^48^, ^50^, ^53^, ^60^, ^61^, ^66^, ^67^, ^70^, ^71^, ^73^, ^76^, ^77^	27
	Imposed losses	^23^, ^24^, ^27^, ^29^, ^34^, ^35^, ^36^, ^37^, ^38^, ^39^, ^41^, ^42^, ^43^, ^48^, ^51^, ^52^, ^53^, ^54^, ^56^, ^57^, ^61^, ^63^, ^64^, ^70^, ^71^, ^72^, ^73^, ^74^, ^75^, ^76^, ^77^	31
2	Reordering life		
	After effects and support needs	^23^, ^26^, ^27^, ^28^, ^30^, ^33^, ^35^, ^36^, ^39^, ^41^, ^43^, ^44^, ^45^, ^46^, ^48^, ^50^, ^51^, ^52^, ^53^, ^54^, ^56^, ^58^, ^60^, ^61^, ^63^, ^66^, ^67^, ^68^, ^69^, ^70^, ^71^, ^72^, ^73^, ^75^, ^76^	35
	Negotiating the support relationship	^10^, ^23^, ^24^, ^25^, ^26^, ^27^, ^28^, ^30^, ^33^, ^34^, ^36^, ^38^, ^40^, ^42^, ^43^, ^44^, ^46^, ^48^, ^49^, ^50^, ^52^, ^53^, ^54^, ^55^, ^56^, ^58^, ^63^, ^64^, ^65^, ^66^, ^68^, ^69^, ^70^, ^71^, ^76^	35
	Readiness to take control	^10^, ^23^, ^25^, ^26^, ^28^, ^30^, ^32^, ^33^, ^34^, ^35^, ^36^, ^37^, ^41^, ^43^, ^45^, ^47^, ^48^, ^49^, ^53^, ^55^, ^58^, ^59^, ^60^, ^61^, ^64^, ^66^, ^68^, ^69^, ^70^, ^74^, ^75^	30
	Reassessing priorities	^10^, ^23^, ^24^, ^25^, ^26^, ^28^, ^32^, ^33^, ^35^, ^36^, ^37^, ^41^, ^43^, ^45^, ^47^, ^48^, ^49^, ^53^, ^55^, ^59^, ^60^, ^61^, ^66^, ^68^, ^69^, ^74^, ^75^	27
3	Choosing a path		
	Being included and given options	^23^, ^28^, ^29^, ^39^, ^40^, ^41^, ^43^, ^47^, ^51^, ^55^, ^56^, ^57^, ^59^, ^60^, ^63^, ^64^, ^66^, ^70^, ^71^, ^72^, ^73^, ^76^	22
	Knowledge is power	^10^, ^27^, ^29^, ^30^, ^39^, ^40^, ^44^, ^49^, ^50^, ^54^, ^57^, ^60^, ^63^, ^66^, ^69^, ^72^, ^76^	17
	Seeing progress and taking time to accept	^10^, ^23^, ^25^, ^26^, ^28^, ^29^, ^31^, ^36^, ^37^, ^38^, ^39^, ^40^, ^41^, ^45^, ^46^, ^48^, ^49^, ^50^, ^53^, ^55^, ^56^, ^57^, ^58^, ^59^, ^60^, ^61^, ^63^, ^65^, ^68^, ^69^, ^71^, ^72^, ^74^, ^75^, ^77^	35
	I've changed a lot	^10^, ^23^, ^24^, ^25^, ^26^, ^28^, ^32^, ^36^, ^40^, ^43^, ^47^, ^48^, ^53^, ^54^, ^55^, ^59^, ^60^, ^61^, ^63^, ^64^, ^65^, ^67^, ^68^, ^69^, ^71^, ^75^, ^77^	27

**Table 5:** illustrative quotes for themes

No.	Theme	Quote
1	Feeling like a second‐class citizen	
	Being depersonalized	Now I have to wait for somebody to do it for me. It's just not the same. (Wood, 2010, p. 1049) There are limitations on motion and of personal control. I'm still very dependent on my caregivers to help me move to the right places. (Herrmann, 2019, p. 5) I always feel that I'm stuck in this chair, waiting for something to happen. It's really difficult. (Burton, 2000, p. 307) They did talk down to a lot of the other people in my room … my slurring was so bad … they'd just pretend that they didn't understand me. I'd think, yes you do bloody know what I'm telling you. You can tell in my tone. (Wolfenden., 2015, p. 9)
	Profound Loss of Self	[I] couldn't seem to control the thinking and just, just feel hopeless and didn't know how to control things. (Walder, 2017, p. 625). It makes you initially feel as if you're losing control of yourself completely [chuckles] … that is a big shake‐up. To feel that, um a part of you has well, maybe died. That is very challenging. (Timothy, 2016, p. 1570). Decisions were made for me, not by me. It seemed to be assumed that the professional staff ‘knew’ what was ‘best’ for me. The system became more important than the individual. (Lawson, 2008, p. 243) If you appear [that] you can take care of yourself, then people normally will give more credence to opinion … if you are ill or unstable or … problems or whatever, then they're going to wonder about your ability to take care of your own self and your family and therefore whether your opinion is tainted. (Anderson, 2013, p. 825)
	Isolated and Trapped	I through went (sic) big changes, absolutely, I was a pilot before and now I can't even drive. I feel much more dependent on others. (Carulli., 2018, p. 33) It stops all social events. It stops the shopping, it stops doing a lot of things that we normally do. (Finch, 2020, p. 6) For a year I didn't drive. That was horrible … I'm not very good at asking for help either. I had no choice … I had to ask for help. (Burton, 2000, p. 323) After my accident, I didn't want friends because I have aphasia and I don't know how to say words. It was very difficult, and I felt dumb being with other friends. So I didn't want to spend time with other people. (Fraas, 2009, p. 321)
	Imposed losses	I don't feel the same as I used to. A bit like a second‐class citizen because I'm not, I haven't got the capabilities that I used to have, being involved with everything like I used to be, knowing everything that was going on around me. (Conneeley, 2003, p. 443) I am very disappointed and frustrated in my lack of progress in recovery … I can't do the chores; I can't even hammer a nail. (Green, 2009, p. 1196) You know it was them [speech pathologist] who decided what I should do. I felt like I was back in 3rd grade. (Berg, 2017, p. 1127) No, they don't always listen, so I have to swallow my pride … I don't always get what I want anymore’. (Anderson, 2013, p. 825) I was shocked and angry to find that well‐meaning professionals planned to set my goals and that I was expected to passively adjust to their system. (Lawson, 2008, p. 243)
2	Reordering life	
	After effects and support needs	I have very limited ability to be mobile … by the time I get up, wake up in the morning, go to the bathroom and get dressed I'm already exhausted and the day has not begun, so the amount of energy it takes to overcome these mobility issues is probably the hardest challenge for me. (Herrmann, 2019, p. 5). Another big problem is food when you're newly paralysed, is eating, because it's difficult to eat with only one arm, and you can't cut food up … I couldn't spread [butter] because you need two hands. (Kitson, 2013, p. 398) since returning [from deployment], with me, it's become more a combination between the not being able to taste, not being able to smell, along with cognitive issues that have arisen: going to the store with a list of five things in my head, and by the time I come out of the store, I'm lucky if I've got one of them. (King, 2018, p. 126). see my husband wasn't—the better I got the more he couldn't understand how I was feeling because he could just see the physical … he just wouldn't understand that I was really tired and it had to do with the stroke. He couldn't understand … the emotional … I'd just start to cry and he'd go ‘What are you crying for?’ … he couldn't understand that I was having a moment. Yeah. My husband moved out. (Wolfenden, 2015, p. 9)
	Negotiating the support relationship	Peer support gives hope: I've questioned the point of struggling with this [illness] myself … but if you give you get absolutely nothing…. (Jumisko, 2009, p. 2276) When I'm at [the ABI program], I'm a nicer person. But I'm just a normal person with them. We all have our problems, from an accident or a stroke. But when we are all there together everyone completely understands. (Fraast, 2009, p. 320) Every time they [formal carers] come in I have to tell 'em what to do (laughs from others) So I give up! (Boger, 2015, p. 183) I do enjoy that because every Monday, me and the guy from here [support worker], we do grocery shopping, and we pick it up and devise the list, and so I do that and it is independent. (Koller, 2016, p. 5). …getting a carer and I didn't want it, I didn't want it at all, I just wanted to be, kind of, independent. Although I wasn't completely on my own but I didn't want any sort of, carers coming in. (Quinn, 2014, p. 1674)
	Readiness to take control	Last week I got my husband to do all that. Drop the kids at school because I just couldn't. I didn't feel clear enough in my own head to face everyone. I didn't feel quite strong enough to talk about it. (P1) (Finch, 2020, p. 5) I felt in control, I knew I either got up there (the stairs) or I didn't get out of hospital, so it was a case of just doing it. (Jones, 2009, p. 510) It's always been but I can't do it right now. I'm not working. I'm not fit to do this. I can't do this. I really can't do this. So it's sad a bit, but I give it the time and everything. Give it the time and see what happens. (Nalder, 2013, p. 1298).
	Reassessing priorities	My job that I wanted to do changed. I knew it was something with kids, but once I was at the Children's Hospital, I met the child life specialist, and I saw what they did and she helped me a lot so now that's what I want to do. (Carulli, 2018, p. 33) I was born a Roman Catholic … I now believe from the Unitarian is to help one another. I'm a person likes to help one another. So I've been going to the Unitarian, and it's a good one cause you don't have to be there every Sunday, it helps me be more level. (Fraas, 2009, p. 321) It's to perform, to achieve something. And then there is getting some money for it. Getting positive feedback on what you do. And that you have a social network around you at work. (P1) (Johansson, 2015, p. 426) To get something to do and mingle with my colleagues, I've many nice co‐workers, who I like to talk and socialise with. Just to get out of bed, catch the bus, get to work and be where you were before. (Taule, 2015, p. 656) I have a shop … my son now helps to run it but most of the decisions are made by me through communicating with him. All the time I am in constant contact with him and I know how much money he is making each day. If I did not have that shop my wife would have left and I would suffer. (P9) (Kamwesiga, 2016, p. 444)
3	Choosing a path	
	Being included and given options	My behaviour's calmed down something big time. [My psychologist] will tell you that I've calmed down a lot … [I credit this to] her because … a lot of mind over matter techniques. Jack (Gould, 2017, p. 305) I don't like passive therapy at all, I like active or proactive, but where I can really get into myself. (Jones, 2009, p. 513) No, I didn't really know what it was all about. But they thought that now I should do my exercises at home. (P8) (Ringsberg, 2003, p. 24) Maybe … I'm saying too much about what I want us to do. And that might not be good, that I'm doing it. Because she is the experienced one, she's the speech pathologist. If I hadn't listened to her I don't think I would have reached this far, I really don't know what to do in therapy. (Berg, 2017, p. 1127)
	Knowledge is power	I was able to get a lot of things that were important to me, in part by being a stubborn SOB, and two, being resourceful. (Price, 2010, p. 114) They kept stuffing pills at me and I'd say what is that for and they would tell me and I'd say no I don't want that and it would be an argument … as soon as I got control of my own input, then I could control my own attitudes towards that. (Herrmann, 2019, p. 5)
	Seeing progress and taking time to accept	It's a struggle to accept the fact that I'm not the person I used to be, that I can't contribute like I used to. (Green, 2009, p. 1197) I think the hardest part is getting learn to live with your new body. You have to take a rest when your body tells you otherwise you're going to pay for it. (Wood, 2010, p. 1051)
	I've changed a lot	I will never go back to the old [me]. That [person] died when they did the operation. I'm a whole new person, I'm more able and more stronger than I never thought I would get. (P3) (Fraas, 2009, p. 322) I don't blame the driver at fault, that doesn't give me nothing to blame him … complaining about what happened is a waste of time. (Dumont, 2007, p. 52) I wasn't going to let anything stop me from succeeding … I had a goal and I said this is the goal that I'm going to accomplish, I'm going to achieve and I'm going to conquer, and I did. (MC) (Kusec, 2020, p. 1333) My life now is completely different from the one I had before. (Artzen, 2014, p. 1629)

### 3.5 Theme 1: ‘Feeling like a second‐class citizen’^37^

Participants reported considerable loss and change in their lives in response to their ABI. This loss had them feeling depersonalized (and second‐class), isolated and trapped as they worked to readjust their sense of self.

#### 3.5.1 Being depersonalized

The sudden impact of the ABI on cognition and function contributed to participants' relinquishing control to others.^49^, ^50^, ^51^ It was difficult for participants to see that they had any control over their recovery or progress.^25^, ^28^, ^53^, ^57^ They became part of a ‘system’ where decisions were made on their behalf,^26^ with an assumption that others ‘knew what was best’,^67^ leaving them feeling overwhelmed,^29^ moody^27^ and depersonalized. When decisions were made without them, they did not know the goals of their therapy, leading to disengagement from rehabilitation.^37^, ^70^ Others ‘swallowed my pride’^66^ and continued with therapy despite their personal goals being unrecognized^66^, ^70^ or feeling they were ‘back in 3rd grade’.^70^ This depersonalized experience diminished their self‐worth^46^, ^58^, ^76^ leaving them feeling like a ‘second‐class citizen’.^25^ This problem was heightened for participants with communication difficulties who felt less like a person without their voice.^44^, ^58^

Depersonalization was accompanied by the awareness of being dependent on and needing to trust others due to physical and cognitive limitations.^27^, ^29^, ^31^, ^35^, ^44^, ^46^, ^57^, ^60^, ^71^, ^77^ This loss of control was described as ‘very frustrating’,^31^ ‘the hardest thing’ (P1),^71^ a ‘disaster’ (P10)^71^ and ‘derailing’.^29^ There were feelings of rage, resentment, fear,^25^, ^58^ helplessness^32^, ^51^ and vulnerability.^43^ Participants struggled with being projected into passive roles where they felt like ‘more of an object for caring measures’^2^ and ‘a vegetable’.^35^ They resented others having to ‘do things for me (them)’,^48^, ^61^, ^71^ or ‘just let(ting) things happen’^44^ and having little choice or freedom.^37^, ^58^, ^60^ There were reports of participants feeling distressed, embarrassed and unkempt.^10^, ^32^, ^53^, ^61^

#### 3.5.2 ‘Profound loss of self’^67^

Dependence limited control over their choices and in turn created profound losses for performing their valued roles^27^, ^37^, ^49^, ^50^, ^53^, ^57^, ^63^ including contribution to society,^48^ relationships,^34^, ^46^, ^63^ custody of children^25^ and having a job/income.^58^, ^74^ There was psychological trauma associated with the incident itself, such as a participant whose child died in a car accident,^25^ or having been the victim of an assault.^25^ Feeling ‘down’, hopeless, depressed, chaotic^76^ or in a dark place^25^, ^29^, ^37^, ^40^, ^59^, ^73^, ^75^ was common.

Identity was challenged as participants contrasted their current situation and new selves after ABI with their former lives.^28^, ^29^, ^37^, ^38^, ^48^, ^52^, ^57^, ^69^, ^73^ A changed sense of self meant participants needed to get to know themselves again.^25^, ^73^ This process of coming to terms with a changed self^25^, ^60^ was described as shocking,^29^, ^32^ traumatic,^76^ distressing,^50^ confronting,^25^ confusing^60^ and emotionally charged.^50^, ^76^ Participants were working on regaining control over their emotions and decisions^26^, ^33^, ^58^, ^73^, ^77^ whilst managing vulnerability.^41^ Apprehension arose about constructing a future that sustained their needs, prior ambitions and familial roles^46^, ^49^, ^57^, ^63^, ^73^ which brought about some grief.^73^

Stigma from ‘being classified as this head injured patient’^47^, ^72^ and fear from feeling out of control^73^ was an ongoing worry; as was lack of knowledge,^36^ which created problems with regaining control.^26^, ^29^, ^43^, ^66^, ^72^, ^74^ Participants described a need to try and prove others wrong,^26^, ^58^ cover up limitations or not disclose problems to keep credibility in the eyes of others.^66^, ^74^ Impairments also affected participants' ability to integrate into social and work situations, leading to worry about appearing different,^59^ and leaving participants feeling ‘othered’.^72^

#### 3.5.3 Isolated and ‘trapped’^28^

Feeling like a second‐class citizen was one consequence of feeling isolated or trapped from impingements on freedom.^43^, ^47^, ^60^, ^63^, ^69^, ^71^ Social integration was an issue^34^, ^43^, ^45^, ^72^, ^75^ due to fear,^32^ loss of confidence,^32^ body changes,^35^ transport limitations,^29^, ^43^, ^47^, ^63^ financial struggles,^29^, ^58^, ^63^ feeling uncomfortable with others,^72^ difficulty speaking,^28^, ^29^, ^32^, ^56^ difficulty remembering people,^28^ feeling that others could not empathize,^28^, ^32^ others being too busy^45^ and imposed medical restrictions.^34^ Decreased social relations and diminished opportunities^34^, ^38^, ^44^, ^76^ were isolating,^26^, ^27^, ^29^, ^39^, ^43^, ^45^, ^50^, ^58^, ^63^, ^76^ ‘suffocating’^29^ and sometimes led to boredom.^32^ Some deliberately chose to avoid people and groups,^28^, ^32^ leading to a smaller circle of social contacts.^29^, ^49^ There was often a loss of choice about when, where, and how these activities occurred.^32^, ^37^, ^38^, ^43^ Restricted choice in social activities caused frustration, stress, dissatisfaction, anger.^31^, ^34^, ^37^, ^46^, ^49^, ^60^, ^69^

#### 3.5.4 Imposed losses

Participants felt like second‐class citizens when they experienced losses from being controlled by institutional systems^26^, ^34^, ^35^, ^36^, ^64^, ^67^ where therapy decisions were made based on the routine practice of the agency rather than individual needs.^42^, ^56^, ^58^, ^61^, ^66^ They reflected that when recovering in hospital, they ‘couldn't leave when I [they] wanted to’,^64^ were in ‘jail’^36^ and ‘not my own person’.^36^ Health professionals who did not listen to requests or consider personal goals in therapy planning reduced autonomy.^42^, ^54^, ^61^, ^66^, ^67^, ^70^, ^73^, ^76^ Choices about service delivery were limited^56^, ^63^ meaning no preference for carer gender,^53^ cultural needs not being met^63^, ^68^ and loss of ability to exercise personal routines.^43^ Health professionals in gatekeeper roles held power over the person with ABI to ‘protect them from harm’^37^, ^75^ which was perceived by some as ‘arrogance’.^67^

Dissatisfaction with rehabilitation and support options^26^, ^59^, ^74^ arose when employers, insurance agencies, family^34^, ^68^ or educators^74^ took over choice and control. For example, it was reported that occasionally restrictive ‘rules’ were set up by family that participants were expected to follow.^41^, ^43^, ^69^, ^71^ Furthermore, legal requirements to take away freedoms were not always managed sensitively, compounding feelings of disempowerment.^30^, ^34^ Examples of disempowerment included lost control over finances,^30^ being prohibited from working with children,^25^ revoked drivers’ licence,^76^ difficulty voting in elections^47^ and imposed restrictions on alcohol consumption or operating machinery.^69^ Where people with ABI did not agree with restrictions placed on them, they sometimes resisted by acting out,^33^ trying to break free or deliberately breaking the rules.^50^, ^69^

### 3.6 Theme 2: ‘Reordering life’^77^

Participants reclaimed their choice and control after their ABI by reordering their life. Participants reframed their understanding of what control meant within their adjusted abilities and choices. This adjustment took place in the context of carer support, peer support and health professional involvement—all of which could be positive but were sometimes counterproductive to the goals and priorities of the participant. Reordering life relied on the capacity to have support needs met, to negotiate the support relationship, and the readiness to take control and prioritize.

#### 3.6.1 After‐effects and support needs

Participants experienced ‘unpredictable’^35^ and often ‘invisible’^30^, ^57^ after‐effects following their ABI.^25^, ^26^, ^28^, ^29^, ^31^, ^32^, ^34^, ^35^, ^39^, ^40^, ^41^, ^42^, ^47^, ^52^, ^53^, ^57^, ^58^, ^59^, ^61^, ^63^, ^65^, ^69^, ^72^, ^73^ Those with communication issues felt particularly constrained in their choices due to difficulty expressing their needs.^53^, ^55^, ^58^, ^77^ To reorder their lives with these after‐effects, participants needed stability^43^ with a gradual reduction in supervision coupled with offering more control over their choices.^34^, ^77^ Counter to this perceived support need, some participants described provided support as excessive, controlling,^26^, ^34^, ^46^, ^57^, ^64^, ^69^ negative deficit focused^26^, ^29^, ^44^ and as prioritizing risk management.^66^ Reordering of life was impeded by inconsistencies with staff, unpredictability of services to meet support needs^36^, ^58^ and inadequate rehabilitation.^27^, ^31^, ^42^, ^43^, ^56^, ^63^, ^77^ Participants perceived that these impediments were inhibiting recovery^25^, ^76^ and identified gaps in services for younger people,^58^ for those living rurally^63^ and those with cognitive and psychosocial issues.^58^, ^76^ To address perceived gaps in their choice of support, participants set up their own rehabilitation programmes,^32^, ^63^ support groups,^73^ sought a second opinion^74^ and sourced psychological services to assist with depression and emotional management.^29^, ^76^

#### 3.6.2 Negotiating the support relationship

Reordering lives required relationships with empowering people^10^, ^26^, ^61^, ^62^, ^66^ who provided time and a positive environment to boost confidence and promote success with community integration.^10^, ^26^, ^51^, ^52^, ^56^, ^62^, ^72^, ^74^ Health professionals helped participants reorder their life by providing encouragement and support.^37^, ^41^, ^44^, ^75^ Successful therapy relationships were collaborative with health professionals working together,^76^ being strength‐focused and curious^33^ about enablers to ‘the process of reordering their [participant] lives and establishing different priorities and goals’.^37^ Support from peers with ABI assisted choice and control because they imparted knowledge, empathy,^25^, ^51^, ^56^ fostered hope,^10^ gave ideas and techniques,^28^ gave reassurance^41^ and a second opinion^25^, ^41^ often without judgement and stigma.^51^, ^62^, ^64^, ^77^

The nature of relationships before the injury and the pre‐existing level of trust and understanding were pivotal.^35^, ^41^, ^63^ Mutually agreed boundaries could be set to keep the situation stable^41^, ^77^; for example, the participant having control over finances but agreeing not to purchase alcohol or spend over a certain amount.^30^, ^41^ Occasionally problems arose, leading to others taking back control^26^, ^30^; this could cause resentment and strain on the support relationship.^29^, ^30^, ^44^, ^54^, ^57^, ^65^

The process of reordering life often involved external services to supplement the care provided by families.^31^, ^54^, ^78^ Some participants were against external support^46^ but others looked favourably on being independent of family carers.^30^ Service systems were lacking,^25^, ^38^, ^58^, ^68^ and once sourced, the quality was variable. Some family carers had to monitor the relationships between the participant and external carers,^77^ other participants reported well‐educated external carers who facilitated control.^49^, ^62^ There was a trade‐off between the benefit of having external carers and having to continually explain needs to new staff.^55^

#### 3.6.3 Readiness to take control

Reordering lives was only possible when participants achieved a readiness to take control. Tensions arose between carers and participants when judging readiness for choices and control.^34^, ^58^, ^65^ Readiness timeframes were highly variable depending on stabilizing of ABI after‐effects.^43^, ^62^, ^65^, ^69^ To give themselves time, some participants left control with family carers,^34^, ^43^ were content with this arrangement and had no immediate intentions to regain control.^30^, ^43^, ^57^ Uncertainty about their current abilities led participants to question their readiness to be left alone or go out alone,^69^ drive, climb a ladder,^43^ manage their finances^41^ or use power tools.^43^ This reticence meant relying on others to judge the risk^43^ or decide limits.^65^ Where choices were made by others, participants wanted to be regularly offered the opportunity to reinstate control.^37^, ^75^

Participants deliberately worked toward reclaiming control.^26^, ^55^, ^61^, ^62^, ^73^, ^76^, ^78^ This was characterized by learning when to seek support, and in what situations,^65^ choosing when to disclose,^40^, ^74^ choosing when to withhold information^41^ and looking carefully at their resources and options for controlling their own mindset, rehabilitation goals and priorities.^10^, ^28^, ^29^, ^35^, ^39^, ^44^, ^45^, ^50^, ^51^, ^56^, ^58^, ^62^, ^64^, ^65^, ^73^, ^78^, ^79^ Personality before the ABI seemed to contribute to how participants approached reclaiming control.^26^, ^32^, ^34^, ^62^ There were some who ‘pushed through’ rather than actively managing and listening to symptoms.^74^ Others took control by choosing to use and accept aids, equipment and compensatory strategies.^25^, ^27^, ^44^, ^45^, ^46^, ^55^, ^57^, ^60^, ^62^, ^65^, ^71^ Choosing to focus on recovery and carry on with life roles within their new abilities was a milestone for participants.^25^, ^30^, ^50^, ^51^, ^57^, ^78^ There was a deliberate lean toward self‐awareness and optimism.^32^, ^36^, ^43^, ^44^, ^50^

#### 3.6.4 Reassessing priorities

The experience of reordering life after an ABI often prompted a reassessment of what gives meaning and purpose in life, personal values and what supports recovery.^25^, ^29^, ^32^, ^34^, ^40^, ^47^, ^48^, ^49^, ^50^, ^51^, ^52^, ^57^, ^59^, ^63^, ^65^, ^72^, ^73^, ^78^ There was an associated adjustment in identity and self‐concept about what to do next.^29^, ^40^, ^48^, ^52^ For some, there was a choice to prioritize health, leisure and family and taking initiative in re‐establishing or maintaining social and familial connections.^29^, ^45^, ^49^, ^52^, ^63^, ^65^, ^73^ Another common priority was independence, self‐management and self‐care,^26^, ^46^, ^55^, ^57^, ^61^, ^65^ and a change or strengthening of spiritual belief systems.^32^, ^56^ There was an appreciation of simple pleasures that were previously taken for granted, such as being alive,^29^, ^37^, ^75^ having a job,^10^ nice weather, slowing down,^29^, ^54^ discovering new tools,^65^ carpentry^40^ and being with family.^25^, ^63^ Participant understanding of the value of having roles and being socially connected plus the desire to take control led them to extending themselves to evaluate their limits.^25^, ^32^, ^40^, ^57^, ^77^, ^78^ There was an eagerness to resume activities that were performed before the ABI,^29^, ^34^, ^57^ but the reflective process established that this was not always possible.^40^, ^43^, ^52^

Some participants were positive about their capacity to progress at work or study, even if needing to reduce load; but others re‐evaluated their ambitions in light of living with an ABI.^32^, ^34^, ^39^, ^40^, ^52^, ^56^, ^73^, ^78^ The reclaiming of their former work/study roles in some capacity was validating and gave a positive sense of purpose and belonging^39^, ^40^, ^45^, ^52^, ^57^, ^76^ for those who chose this path. Finding work, volunteering or returning to study were often key steps toward achieving a positive outlook, even when symptoms following the ABI persisted.^10^, ^25^, ^40^

### 3.7 Theme 3: ‘Choosing a path’^25^

Participants consolidated what control over choices meant for them, exerting agency and establishing a stronger understanding of self, and what they do and do not need in their lives to adjust. Participants experienced choice and control when they could choose a path to acceptance because others included them in decision‐making, and provided options, information and time in ways that enabled participants to see how much they have changed.

#### 3.7.1 Being included and given choices

Participants experienced inclusion when they felt respect for their dignity and privacy and when given the opportunity to exercise choice so they could learn, adapt and get to know themselves better.^25^, ^32^, ^47^, ^53^, ^57^, ^69^, ^78^ Where participants were excluded, they felt devalued and discouraged.^42^, ^46^, ^47^, ^58^, ^70^ Inclusion led to hope and confidence^32^, ^40^, ^45^, ^76^; particularly when addressed personally and listened to,^36^, ^42^, ^47^, ^54^, ^67^, ^70^ when consulted about their rehabilitation/health/study plan,^35^, ^40^, ^42^, ^74^ and when part of goal setting.^32^, ^53^ Including the participant in goal setting promoted trust^61^ and was described as a powerful experience.^36^ When participants were provided with choices by health professionals they could choose their path based on their preferences, values and goals^53^, ^64^ even if they needed help.^38^

#### 3.7.2 Knowledge is power^67^

Choosing a path to acceptance in the context of their trauma, diagnosis and adjusted abilities, relied on the participant accessing information and knowledge from health professionals. Having access to knowledge promoted empowerment and security^36^ and in turn, provided relief^10^, ^36^ and hope.^51^ Knowledge was pivotal to participant capacity to regain control^46^, ^51^, ^67^, ^74^ because it gave collateral to ask questions. They wanted information about their condition, medical procedures, medications, prognosis^44^, ^54^, ^60^, ^69^ and time frames for therapy.^70^ There was not always sufficient information forthcoming for the participants to make autonomous and complex decisions about their desired path.^32^, ^38^, ^42^, ^55^, ^74^ Some participants took matters into their own hands through independent research.^42^, ^51^, ^58^ Others persisted with stating their preferences and asked lots of questions to keep control over their interests and push back on decisions they disagreed with.^38^, ^42^, ^51^, ^66^

#### 3.7.3 Seeing progress and taking time to accept

Progress toward acceptance and choosing a path required patience and resilience.^34^, ^35^, ^64^, ^70^ Participants commented on the internal struggle to adjust to limitations on roles and abilities^34^, ^50^, ^56^ and not everyone reached a point of acceptance.^25^, ^29^, ^39^, ^67^ Choosing to maintain hope and persevering were ways for keeping control^35^, ^50^, ^57^, ^62^ and acceptance was discussed in terms of years; with reflections spanning from less than 2 years to 20 plus years. Acceptance was described as a ‘freeing’ experience^29^ with a re‐evaluation of values^29^, ^34^, ^38^, ^52^, ^57^ and adoption of self‐care.^32^, ^34^

Seeing progress was motivating^10^, ^26^, ^36^, ^51^, ^64^, ^71^, ^78^ and often centred around new ways of doing things,^10^, ^34^, ^35^, ^39^, ^49^, ^57^, ^60^, ^62^, ^63^, ^65^, ^73^, ^74^, ^78^ establishment of a routine for self‐regulation and self‐management,^10^, ^25^, ^28^, ^29^, ^33^, ^52^, ^77^ systematic planning (i.e., writing things down; reminders on their phone; planning travel routes and meals)^10^, ^25^, ^47^, ^52^, ^57^, ^65^ and sleep preservation.^25^ Progress on the chosen path included prioritizing what were nonnegotiable ‘musts’ in their lives, what was flexible^10^, ^29^, ^63^ and accepting that plans may need to change.^35^, ^39^, ^52^, ^73^

Regaining control was further marked by the re‐establishment of paths that were perceived as ‘normal’.^40^, ^50^ The path was accepted as being individual,^29^, ^34^, ^40^, ^57^, ^73^ and included activities like having a beer with friends,^35^ going to the shops,^32^ making adjustments to return to study or work^25^, ^39^, ^40^, ^42^, ^52^, ^59^, ^74^ and being independent with daily activities.^44^, ^49^, ^65^, ^78^ This also included participants returning to past leisure activities such as surfing,^73^ hunting, skiing^25^, ^62^ or valued roles.^29^, ^57^, ^77^

#### 3.7.4 ‘I've changed a lot’^48^

With this process of choosing a path, there was a realization to ‘make the most of the situation’^62^ and aim for growth in self‐confidence, purpose and autonomy.^25^, ^26^, ^29^, ^57^ Acceptance also meant finding the strength to work out what to do now^10^ often leading to accomplishments, such as library work,^57^ scrapbooking,^78^ photography,^62^ driving, dancing or regaining a valued role.^10^, ^56^ Gains (physical, psychological and cognitive) enabled productive and fulfilled lives—oftentimes giving back to the community.^10^, ^25^, ^29^, ^32^, ^38^, ^48^, ^56^, ^57^, ^61^, ^73^, ^74^ There was perseverance and pride in the progress made,^44^ going from feeling overwhelmed to regaining control through seeking independence ‘to go where I want, when I want’^71^ (P1).

The initial loss of both roles and independence resulted in a reframing of identity and priorities.^37^, ^38^, ^49^, ^57^ They aimed to make choices that aligned with their values, and this started to shape their perception of who they were and wanted to be.^26^, ^29^, ^40^, ^56^, ^57^, ^65^, ^74^ Participants were mindful of how the ABI may have modified their personality, priorities and abilities.^29^, ^32^, ^34^, ^48^, ^52^, ^57^, ^63^, ^65^ For some it meant altering their lifestyle, focusing on healthy choices and putting effort into valued occupations (i.e., study).^32^, ^40^, ^48^, ^54^, ^63^ Some participants felt more content when they lived in the moment without placing pressure on themselves,^10^, ^29^, ^34^ others chose to be forward thinking^25^, ^30^, ^32^, ^74^ at the same time as being kind to themselves.^29^, ^32^, ^37^, ^71^ Participants began to identify with being a person who is dependent on others,^26^, ^27^, ^65^, ^73^ who has constraints on ability^29^, ^37^, ^56^, ^57^ and who has a changed future and relations.^25^, ^45^, ^57^, ^61^ With increasing control and flexibility over their choices and opportunities, participants became more future‐oriented.^26^, ^29^, ^35^, ^37^, ^71^, ^74^

## 4 DISCUSSION

This meta‐synthesis of 56 papers involving 765 participants with ABI explored their perspectives of choice and control in daily life while living in the community. Three interdependent themes were generated. The first (Theme 1—feeling like a second‐class citizen) is the person struggling with the loss of choice and control in a highly vulnerable state. The second (Theme 2—reordering life) is coming to terms with the after‐effects of ABI, support needs and priorities for the future. The third (Theme 3—choosing a path) is making decisions about identity and the future. Choice and control are initially lost after ABI and regaining them can both result from, and drive, positive recovery in an external sense and from an internal or individual point of view.

The tensions between expert and supported decision‐making in theme one arise from the inherent loss of choice and control as the person emerges from the ‘patient’ role where the model of care and service may be seen as expert‐led and disempowering, albeit lifesaving. The resulting impairments, activity restrictions and limitations initially become apparent in the controlled hospital environment where dependencies become manifest. However, it is also apparent that the transition out of acute medical care (and the requisite safety considerations) can be slow or incomplete, whereas people following ABI need it to be dynamic to allow the emergence of their choice and control from these dependencies in an explicit and staged manner tailored to their needs. As the impact of the ABI becomes more understood and accepted by individuals, there needs to be a parallel conversation about how these dependencies can best be approached so that even, amid dependency, people can begin to have choice and control. This sensitivity requires constant mindfulness from health professionals who implicitly hold power in post‐ABI care; from timetabling, services, approaches and goal setting^80^, ^81^ even through to hope.

The simple yet highly effective approach of collaborative goal setting *from the person's point of view* is one of the most evidence‐based ways to ensure that the person feels they are at the centre of choices and control.^82^, ^83^ Offering choice in how best to work towards these goals could be a part of service delivery that is negotiated rather than prescribed. Structured frameworks for shared decision‐making could be explicitly applied to support negotiations and collaborations.^18^ Tacit discussions about risk identification and mitigation between the person, their health professionals and family/supporters again shift the power to a shared model.^9^ Empowerment can be explicitly staged into rehabilitation pathways with education, understanding, responsibilities and decision‐making discussed and shared.

This leads to needing dynamism between protection from harm and the opportunity to test limits that occur as the person widens their environment to include family, social and vocational domains (Theme 2). Again, dependencies need to be negotiated so that choice and control can occur in a staged way—from the timing of activities to the amount of assistance and degree of participation. This requires the broader support network to become part of the negotiation and education including family, friends, co‐workers and supporters.^84^

Finally, in Theme 3, the meta‐synthesis has identified the need for staged, bespoke and deliberate restoration of choice and control for people following ABI (rather than one approach for all). This need arises when the person with ABI is ready to exercise choice and control to renegotiate their new path. This renegotiation involves deciding where and how they find meaning and purpose and make sense of their changed abilities. Community‐based services could pivot to this goal or series of goals and enable individuals to discuss and explore internal themes of agency and self‐determination by changing the locus of control and enhancing self‐efficacy. Evidence is emerging that programmes for people with ABI that are based on self‐management can improve self‐efficacy^85^ and empowerment can emerge from staged mastery and competency processes.^86^

### 4.1 Strengths and limitations

The review had a preregistered protocol and followed gold standard reporting (ENTREQ statement).^20^ Screening, data extraction and critical appraisal were completed independently by two reviewers. Most papers are from developed countries, thus limiting the findings to these cultures. Grey literature was not included, which may have excluded valuable nonpeer‐reviewed information. Concerns about the absence of positionality of authors within included papers may be offset by consistent reporting of participant quotes in the papers and a large number of studies. In addition, we approached the study with sensitivity and took steps to ensure rigour and reflexivity in the interpretation of the findings. Due to the review occurring over a 7‐year period (2016–2022) and the lack of funding to appropriately support consumer engagement, we did not have a person with lived experience on the research team.

### 4.2 Recommendations and conclusion

People with ABI describe a dynamic and often problematic process of re‐engaging with choice and control after their life‐changing injury. Health professionals and supporters need to be equipped with the skills and knowledge to facilitate a gradual and negotiated return to agency for people with ABI.^87^ These skills include being collaborative, focused on the individual with ABI and knowledgeable about dignified risk management.^9^ Clear service or process indicators of change need to be developed to allow for evaluation and monitoring of services such as:

(1) participant inclusion in goal setting,
(2) explicit conversations to negotiate choice and control between health professionals, people with ABI and their supporters,
(3) education for health professionals about the complexities of informed choice, control, empowerment, risk, support and advocacy,
(4) tacit sessions to address understanding, acceptance and competence individually and in peer groups,
(5) education for the community and social supporters that raises awareness of the issues described by people with ABI (and helps them develop the skills to support the person).

Recommendations for practice include regular ‘checking’ in with the person with ABI about their readiness to engage with choice and control, a tailored approach to health professional involvement and collaborative involvement in setting rehabilitation goals as an imperative.

## AUTHOR CONTRIBUTIONS

The project was led by Carolyn M. Murray with the grant CI and the senior author being Mandy Stanley. All authors were involved at different stages of the research and provided intellectual input in the study design. The authors involved in database searching were Scott Weeks, Michelle Guerin and Emma Watkins. The authors involved in screening were Carolyn M. Murray, Scott Weeks, Gisela van Kessel, Michelle Guerin, Shylie Mackintosh, Caroline Fryer and Mandy Stanley. Authors involved in data extraction were Carolyn M. Murray, Michelle Guerin, Gisela van Kessel and Emma Watkins. Authors involved in critical appraisal were Carolyn M. Murray, Gisela van Kessel, Emma Watkins and Caroline Fryer. The authors involved in data synthesis were Carolyn M. Murray, Scott Weeks, Emma Watkins, Michelle Guerin, Gisela van Kessel, and Mandy Stanley. The authors involved in manuscript preparation were Carolyn M. Murray, Michelle Guerin, Mandy Stanley, Scott Weeks, Shylie Mackintosh, and Susan Hillier. All authors contributed to manuscript editing and approved the final manuscript.

## CONFLICT OF INTEREST

The authors declare no conflict of interest.

## Supporting information

Supporting information.

Supporting information.

Supporting information.

## Data Availability

The data that support the findings of this study are available from the corresponding author upon reasonable request. The review is secondary research. All data used in the review is available in the included published papers.
