# Supplementary material for: Perspectives of choice and control in daily life for people following brain injury: A qualitative systematic review and meta‐synthesis
Source: Health Expect. 2022 Oct 31;25(6):2709–25. doi: 10.1111/hex.13636 (PMC9700193; doi:10.1111/hex.13636)
Supplement: Supplementary file 1 — Supporting information. [file HEX-25--s003.docx]

**Supplement 1: Search terms and example search string in Medline**

1. Choice Behavior/
2. Decision Making/
3. Personal Autonomy/
4. (personal OR individual* OR autonom*) AND (choice OR decision).mp
5. 1 OR 2 OR 3 OR 4
6. (personal OR individual* OR autonom*) AND (control* OR power OR determinat*).mp
7. 5 OR 6
8. Brain Injuries/
9. traumatic brain injury.mp
10. head injury.mp. OR Craniocerebral Trauma/
11. Stroke/
12. cerebrovascular accident.mp
13. cerebral vascular accident.mp
14. cerebrovascular insult.mp
15. 8 OR 9 OR 10 OR 11 OR 12 OR 13 OR 14
16. 7 AND 15
17. (personal OR individual* OR autonom*) AND (view* OR viewpoint* OR opinion* OR thought* OR understanding* OR perspectiv* OR perception* OR outlook OR feeling* OR belief* OR considerat* OR experienc*).mp
18. 16 AND 17
19. limit 18 to (humans and yr="1980 -Current")

| **Database** | **Search no.** | **Search terms** | **1980-2016** | **2016-2022** |
| --- | --- | --- | --- | --- |
| **Medline (R)** | 1 | Choice Behavior/ | 32997 | 67199 |
|  | 2 | Decision Making/ | 97123 | 198395 |
|  | 3 | Personal Autonomy/ | 17269 | 35171 |
|  | 4 | (personal OR individual* OR autonom*) AND (choice OR decision).mp | 107356 | 224513 |
|  | 5 | 1 OR 2 OR 3 OR 4 | 218471 | 450930 |
|  | 6 | (personal OR individual* OR autonom*) AND (control* OR power OR determinat*).mp | 607133 | 1262451 |
|  | 7 | 5 OR 6 | 790892 | 1640899 |
|  | 8 | Brain Injuries/ | 52505 | 106402 |
|  | 9 | traumatic brain injury.mp | 38481 | 80326 |
|  | 10 | head injury.mp. OR Craniocerebral Trauma/ | 34475 | 69941 |
|  | 11 | Stroke/ | 105911 | 222163 |
|  | 12 | cerebrovascular accident.mp | 4697 | 9702 |
|  | 13 | cerebral vascular accident.mp | 612 | 1241 |
|  | 14 | cerebrovascular insult.mp | 163 | 329 |
|  | 15 | 8 OR 9 OR 10 OR 11 OR 12 OR 13 OR 14 | 207911 | 431304 |
|  | 16 | 7 AND 15 | 10095 | 21129 |
|  | 17 | (personal OR individual* OR autonom*) AND (view* OR viewpoint* OR opinion* OR thought* OR understanding* OR perspectiv* OR perception* OR outlook OR feeling* OR belief* OR considerat* OR experienc*).mp | 498011 | 1045312 |
|  | 18 | 16 AND 17 | 2308 | 4825 |
|  | 19 | Limit 18 to (humans and yr=“1980 – Current”)  Limit 18 to (humans and yr=“2016 – Current”) | 757 | 879 |
