# Supplementary material for: Perspectives of choice and control in daily life for people following brain injury: A qualitative systematic review and meta‐synthesis
Source: Health Expect. 2022 Oct 31;25(6):2709–25. doi: 10.1111/hex.13636 (PMC9700193; doi:10.1111/hex.13636)
Supplement: Supplementary file 2 — Supporting information. [file HEX-25--s002.docx]

Supplement Appendix S2 Characteristics of included studies

|  | Author, year country | Aim | Methodology | Participant and public involvement | Data collection | Participants | Type of ABI | Time since injury | Relevant study findings |
| --- | --- | --- | --- | --- | --- | --- | --- | --- | --- |
| 1 | Allen  2021  Ireland | Experience of living with brain injury according to personal growth | Critical realist | Interview guide piloted | Survey followed by single semi-structured interviews | 14µ; 8 m, 6 f.  Mean age = 47 | 7 TBI; 5 stroke; 1 encephalitis; 1 brain tumour | Mean 7.88 years  Range = 7.22 – 8.72 years | Acceptance, self-integration and social connection support personal growth after brain injury |
| 2 | Anderson 2013 Canada | Understand resources that enhance activity participation after stroke | Grounded theory approach | No | Single one hour interviews | 9; 6 m, 3 f.  Age range 53 – 64 | All stroke | Date of stroke given but date of data collection unknown | Changes in how viewed by others requiring continual negotiating and positioning for regaining and retaining control |
| 3 | Arntzen 2014  Norway | Long term negotiations and recovery trajectory | Not given  Longitudinal  Phenomenology for analysis | No | Multiple in-depth interviews | 9; 6 m, 3 f. Age range 39 - 72 | All stroke | 6 months – 12 years (6/9 over 5 years) | Progress is seen as renewed relationships, understanding their body, participation in activities and transformed self |
| 4 | Berg 2017  Norway | Participation in goal setting with aphasia | Not given | No | Semi-structured interviews | 15; 7 m, 8 f.  Age range 43 – 74 median 61 | All stroke | 4 – 116 months  Median 19 | Prefer being included in conversation and feeling heard. May withdraw if not. |
| 5 | Boger 2015 UK | Explore factors facilitating or hindering stroke self-management | Exploratory sequential mixed-methods design | Confirmation sought from people with speech limitations | Qual aspect - Five focus groups | 28; 11 m, 17 f.  Mean age 65.67 | All stroke | Approx. range 23.5 - 83 months  Mean 57.89 | Issues in emotional recovery – loss of control over body, circumstances and social life leading to frustration |
| 6 | Burton 2000 UK | For people to describe their own recovery. | Phenomenology with grounded theory principles - longitudinal | No | interviews conducted monthly over 1 year (73 total) | 6; 4 m, 2 f.  Mean age 67 | All stroke | First interview after discharge and then monthly for at least 1 year | Uncertainty and loss of control over body and circumstances. Emotions – anger, fear. With recovery came hope. |
| 7 | Carulli 2018  USA | Student social engagement | Phenomenology | Member checking | Single interviews | 12; 8 m, 4 f.  Age range 18 – 36 Mean 24.8 | Traumatic brain injury | Range 1.5 – 5 years  Mean 2.9 (SD 1.2) | Change in attitude, priorities interests; new identity and big life changes |
| 8 | Conneeley^Ϯ^ 2002 UK | Explore social integration issues following rehabilitation | Interpretative, Phenomenology, Longitudinal | Member checking | Multiple individual interviews over 1 year following discharge | 18;13 m, 5 f. - Mean age 35  Range  17 - 60 ≠ | Severe traumatic brain injury | Interviews at discharge, 6 months and after 1 year. Time in hospital varied 1 month – 12 months | Reclaiming personal autonomy and control was central to identity, meaning and purpose. |
| 9 | Conneeley^Ϯ^ 2003 UK | Quality of life after rehab | Interpretative, Phenomenology, Longitudinal | Member checking | Multiple individual interviews over 1 year following discharge | 18;13 m, 5 f. Mean age 35  Range  17 - 60 ≠ | Severe traumatic brain injury | Interviews at discharge, 6 months and after 1 year. Time in hospital varied 1 month – 12 months | Quality of life was sense of wellbeing, functional status, personal autonomy and acceptance of disability |
| 10 | Conneeley^Ϯ^ 2012 UK | Explore the transition from hospital to home | Interpretative, Phenomenology, Longitudinal | Member checking | Multiple individual interviews over 1 year following discharge | 18; 13 m, 5 f. Mean 35  Range  17 - 60 ≠ | Severe traumatic brain injury | Interviews at discharge, 6 months and after 1 year. Time in hospital varied 1 month – 12 months | Return to normal/ work, lacking information, emotional. Lacking control in decisions / family crucial |
| 11 | Dumont 2007  France  (translated) | Adaptation process and coping strategies | Phenomenology | Interview guide development | Single semi-structured interview | 53; 37 m, 16 f.  Mean age = 37.5 | Traumatic brain injury | Completed rehab between 1 and 5 years prior | Resumption of occupations is central, along with resolving emotional upheaval and acceptance new self |
| 12 | Finch 2020  Australia | Experience following minor stroke | Qualitative Descriptive | No | Semi-structured interviews | 17; 12 m, 5 f.  Mean age = 68.29 | Minor stroke | Mean 17 days since hospital discharge* | Control often relinquished to others. Others advising on choices; being self-protective |
| 13 | Fraas 2009  UK | Factors for successful recovery and lifestyle | Phenomenology approach with case studies | People with ABI conducted interviews  Member checking | Single Semi-structured life story interviews | 31; 21 m, 10 f. Mean age = 44 | 16 traumatic brain injury; 11 stroke | Mean 133.5 months | Accepting asking for help, developing confidence again, adjusting to new abilities and re-establishing priorities |
| 14 | Gallagher 2011  Canada | Process of emotional recovery | Grounded theory | Member checking | Single unstructured interviews; 1 focus group | 9; 5 m, 1 f, 3 unknown.  Age range 42 – 82 | All stroke (3 with aphasia) | Range 6 months – 4 years (median 1 year) | Distress, fear, deliberate decision to rehab, naming and prioritising valued skills, persevering and taking risks |
| 15 | Gould 2019  Australia | Experience of behaviours of concern | Not given | Member checking and stakeholders meeting | Single semi-structured interviews | 11m £  (4 eligible β)  Age range 29 – 56,  mean = 44.2 | Traumatic brain injury | Range 1 – 35 years  Mean 14.8 | Realising social behaviours self-done with professional support. Meaningful activities and routine were valuable |
| 16 | Graff 2020  Denmark | Barriers and facilitators to returning to work | Not given  Exploratory | No | Single semi -structured interviews | 22; 8m, 14 f. Age range 24 - 60 | Traumatic brain injury | range 20–60 months  Mean 33.5 months | Workplace accommodations were lacking; follow up assessments need to be more targeted and helpful |
| 17 | Green 2009  Canada | Impact of quality of life | Not given  Longitudinal, descriptive | No | Number not given – 6 interviews with each participant = 156 | 26 m.  Age range 39 – 83 (also stated 48 – 82 years)  Mean = 64 | Minor stroke | 12 months after discharge* | Disrupted sense of self, feeling vulnerable and frustrated, learning to adapt, new perspectives, valued relationships |
| 18 | Häggström 2008  Sweden | Experience of participation in daily life | Not given  Exploratory | No | Single interview | 11; 5 m, 6 f.  Age range 38 -  62. Mean 55 | 10 stroke, 1 traumatic brain injury | Range 3 – 6 years | Choice and expressing wishes essential, needed support, options and information. Aimed to contribute |
| 19 | Hammond 2021  USA | Explore Political participation after brain injury | Participatory research approach  Member checking  Grounded theory analysis | Stakeholders consulted throughout research | Interviews and field observations of voting | 57 £ (54 eligible µ);  41 m, 16 f.  Mean age at injury 41.2 | Traumatic brain injury | Not reported | Voting represents freedom and voice but can be limited |
| 20 | Harrington 2015  Australia | Experiences of pathways, outcomes and choice | Not given | Member checking | Single Semi structured interviews | 10; 9 m, 1 f.  Age range 20 - 50 | Severe traumatic brain injury | Range 8 months – 19 years | System limits choice and inaccurate at times. There exists uncertainty and lack of information about options. |
| 21 | Harris Walker  2021  USA | Influences on recovery for younger adults | Longitudinal, convergent mixed methods design | No | Single semi-structured phone interview | 20 interviewed  Median age 54  All under 65 years £  Gender unknown | Stroke | 3 months | Recovery effected by depression, fatigue, family, social supports, working,  insurance and income |
| 22 | Herrmann 2019  USA | Experiences of hospitalisation and recovery | Descriptive Qualitative | No | semi-structured interview | 6; 2 m, 4 f.  Age range 65 – 80 | Traumatic brain injury | Admitted in the last 2 – 6 weeks* | Uncertainty, shock, fear, loss of control, physical limitations, support was needed |
| 23 | Johansson 2016  Norway | Daily activities and roles for returning to work | Grounded theory | Member checking | 24 semi-structured interviews | 8 m  Age range 30 – 60 | Traumatic brain injury | At third interview – Range 20 – 28 months | Control for when to return to work and accepting adjustments made, ambitious, and self determined |
| 24 | Jones 2008  UK | Personal factors and resources to support recovery | Phenomenology approach | Pilot interview with 5 people  Member checking | Free-flowing non-directive single interviews | 7β; 5 m, 2 f.  Age range 29 – 7  Mean 59.4 | All stroke | Range 2 – 13 months  Mean 7.3 months | Optimism, motivation, active in therapy, skills, roles, self-sufficiency, being home |
| 25 | Jumisko 2009  Sweden | The meaning of feeling well | Phenomenology hermeneutic interpretation | Pilot interview with one person | Single Interviews (concrete, direct) | 8; 6 m, 2 f.  Age range 29 – 53  median 41 | Moderate or severe traumatic brain injury | Range 7 – 15 years  median 10 years | Relationships, positive mind-set, prioritising what they valued, feeling useful, having a routine |
| 26 | Kamwesiga 2016  Uganda | Experience of using mobile phones | Grounded theory | No | Single semi-structured interviews | 11; 6 m, 5 f.  Age range 25 – 75 years | Stroke | Range 7 months - 20 months | Mobile phone enables communication, agency, routine, well-being, security |
| 27 | Kelly  2021  Australia | Rehabilitation for Aboriginal people | Not given  Qualitative | Aboriginal people on research team | semi–  structured interviews | 6 (4 eligibleβ): 3 m; 1 f.  Age range 30-69 | Stroke | Previous 3 years | Communication; involvement of family, co-morbidities, relationship with services |
| 28 | Kessler 2013  Canada | The process of change during recovery | Grounded theory | No | Single Semi-structured interviews | 12; 10 m, 2 f.  Age range 44 – 74  mean 54 | All Stroke | Range 3 – 11 years  mean 6.5 years | Vulnerability, choosing recovery, connected to others (peers), knowledge = hope & motivation |
| 29 | King 2018  USA | Factors that inform beliefs | Qualitative part of Mixed methods | No | Single Semi-structured interviews | 22; 19 m, 3 f.  Mean age 45  Between ages 18 and 65 | Traumatic brain injury | Not reported | Moderate activity to avoid feeling overwhelmed. Loss of physical and cognitive control |
| 30 | Kitson 2013  UK | Experience of fundamentals of care | Interpretive phenomenology | No | Single Narrative interviews | 15; 6 m, 9 f.  Age range mid 30s – mid 80s | All Stroke | Not given (secondary analysis of data) | Dignity, communication, privacy, education, respecting choice (male carers) |
| 31 | Knox 2016^∞^  Australia | Understanding decision-making | Constructivist grounded theory | Member checking | 18 π multiple in-depth interviews | 4; 3 m, 1 f.  Age range 27 – 47  Mean 35.5 | Severe traumatic brain injury | Range 7 – 17 years | Trust, collaborative, evaluation of risk and responsibility, supports, autonomy, information, |
| 32 | Knox 2017^∞^  Australia | Exploring decision making and self concept | Constructivist grounded theory | Prolonged engagement – member checking | Multiple individual in-depth interviews | 7 β; 5 m. 2 f.  Age range 20 - 49 | Mod / severe traumatic brain injury | Range 7 – 19 years | Self-concept strongly linked to having independent or supported choices |
| 33 | Koller 2016  Canada | Experiences of financial management | Qualitative Descriptive | No | Single semi-structured interviews | 6; 5 m, 1 f.  Age range 37 – 51.  Mean 44.5 | 4 traumatic brain injury and 2 stroke | Range 9 – 26 years  Mean 17 years | Struggle between desire for control and accepting supports. Strategies were used by family to support. |
| 34 | Kubina 2013  Canada | Reengagement in activities | Constructivist grounded theory | Informal member checking | 30 individual interviews | 6; 3 m, 3 f.  Age range 40 – 68. Mean 58 | All Stroke | 5 interviews  6, 9, 12,18, and 24 months post stroke | Social connection, being in charge of adaptations, some changes in valued activities |
| 35 | Kusec 2020  Canada | Engagement in community based programs | Interpretive descriptive | Informal member checking | Semi-structured interviews | 21; 18 m, 3 f.  Age range 32 – 64. Mean 47.7 | 14 Traumatic brain injury  7 stroke or infection | Mean 18. 29 years | positive motivators gave rise to a sense of choice  and control, which led to increased engagement |
| 36 | Lawson 2008  Canada | Personal narrative of rehabilitation | Autoethnography | Author is participant | Reflection, journal, poetry, interview | 1 adult woman  Age not specified | Brain injury and poly-trauma | Data collection began 5 years after injury | The ‘system’ took away power and identity. Providing information was helpful but taking over was not |
| 37 | McCluskey 2007  Australia | The process of care management | Grounded theory | Member checking | 14 Single interviews | 14; 8 m, 6 f.  Age range 19–56. Mean 36.5. | Traumatic brain injury | Ranged from 2.5 to 37 years  mean 9.9; median 8.7 | Skill development, goal setting and routines. Risk tolerance supported autonomy. Care decision making was iterative |
| 38 | Mealings  2021  Australia | Student participation | Longitudinal Grounded theory | Member checking | A series of interviews – total of 30 | 9 ¥; 8 m, 1 f.  Age range 18 – 30 | All traumatic brain injury | Ranged 4 months – 4 years | Choosing to study creates a new pathway with key decision making timepoints |
| 39 | Moss  2021  UK | Psychosocial adjustment with aphasia | Not given  Qualitative | Interview guide co- developed | Single semi-structured interviews | 20; 10 m, 10 f. Age range 25 - 85 | All stroke | Randomised when <6 months post stroke, interviews 4 months after that | Humanise service provision, mood, sense of self, outlook, advocacy needed |
| 40 | Nalder 2013  Aus | Experiences of going home | Not given  Qualitative | Member checking | Single interviews | 16; 15 m, 1 f.  Age range 18 - 55 | TBI | 6 months post discharge* | Strive for normalcy through activities and arrive at changed world view |
| 41 | Olofsson 2005  Sweden | Reflections on hospital and going home experiences | Not given  Qualitative | No | Single interviews | 9; 4 m, 5 f.  Age range 64 – 83  Mean 72 | All Stroke | Not specified | Initially depersonalised; yearned to go home. Progress improved confidence |
| 42 | Paniccia 2019  Canada | Transition to work roles | Descriptive qualitative | No | Single interviews | 13; 5 m, 8 f.  ¥  Age range 18 – 25 | 11 TBI  1 stroke; 1 tumour | Range from 1 year to 17 | Negotiating accommodations at work involved disclosure, knowledge and agency |
| 43 | Pereira 2020  Portugal | Perspectives on adaptation over time | Not given  Longitudinal | No | Semi-structured interviews | 8; 6 m, 2 f.  Age range 43 – 79 Mean 66 | All stroke | Two interviews at 1 and 6 months after discharge* | Seeking to regain former lives shifted to making new goals and adapting to new priorities. |
| 44 | Price 2012  USA | Narrative about resilient adaptation | Not given | Member checking and reviewed drafts | 2 interviews one month apart | 1 m  In his 70s | Stroke | 20 years | Resourceful, goal oriented, received personal spiritual support to foster continued identity through occupation |
| 45 | Quinn 2014 UK | Experience of young couples after stroke | Interpretative phenomenological analysis | No | Single interviews with dyad^ | 8; 7 m, 1 f.  Age range 36-65 | All Stroke | Range 1 – 9 years | Couples undergo role disruption and adjustment |
| 46 | Ringsberg 2003  Sweden | Perspectives of home rehabilitation | Phenomenology | No | 6 focus groups | 15; 11 m, 4 f. age range 59 - 85.  Mean 69 | All Stroke | Not specified | Information needed, dependence on transition home, social roles and mood change once home. |
| 47 | Satink 2016  Netherlands | Self  management through everyday activities | Not given  Longitudinal | No | 50 informal interviews plus participant observations | 10; 4 m, 6 f. age range 54 – 77 | All stroke | 5 interviews at 3, 6, 9, 15, 21 months | Learning to self-manage through prioritisation and ‘doing’ contributes to gaining control over life choices |
| 48 | Sveen 2016  Norway | Everyday occupations and return to work participation | Not given | No | 7 focus groups | 20; 8 m, 12 f. age range 22 – 60  Mean 40 | Mild traumatic brain injury | Range 11 – 87 weeks  Median 30 | Adjustment to changes in fatigue, organisation and memory took time and effected participation |
| 49 | Taule 2015  Norway | Experiences of home rehab | Interpretive Description | No | Single interviews | 8; 4 m, 4 f.  Age range 45 – 80 | All Stroke | 6 – 8 months | Rehab not always goal directed. Sought engagement, empathy and equality. |
| 50 | Timothy 2016  New Zealand | Embodiment while transitioning to home | Constructivist Grounded theory | Member checking | 24 semi-structured interviews | 6 β; 5 m, 1 f.  Age range 66 - 84 | All stroke | Weekly after discharge* for 1 month (LOS ranged 4 – 12 wks) | Sense of self, personal attributes, environment, knowledge and attitudes can “anchor” |
| 51 | Tomkins 2013  Australia | Satisfaction with health care | Qualitative descriptive phenomenology | No | Single Semi-structured interviews | 50; 24 m, 26 f.  Mean age 63.9 | All stroke | Mean 54.9 months | Control = decision- making, information provision and inclusion and free will |
| 52 | Turner 2009  Australia | Reengagement in meaningful occupation for youth | Phenomenology | No | semi- structured interviews | 20; 15 m, 5 f.  Age range  17 - 63≠ | 16 traumatic brain injury  4 stroke | 3 interviews Predischarge*, 1 and 3 months post discharge | Frustrated about restrictions and lack of information. Difficulty establishing routine |
| 53 | Vestling 2013  Sweden | Thoughts and feelings about return to work | Not given | No | Single semi-structured interview | 12; 8 m, 4 f.  Age range 43 – 61  Mean = 53 | All stroke | 2 months – 26 months | Returning without expectations, adapting on their terms, being trusted, fatigue, depression |
| 54 | Walder 2017  Australia | Re-establishing occupational identity | Constructivist grounded theory | Member checking | Single informal interview | 6; 2 m, 4 f.  Age range 34 - 76 | All stroke | 4 months – 3 years | Loss of control was scary. Used positive mindset and reprioritisation of life goals |
| 55 | Wolfenden 2015 Australia | Vulnerability of younger high functioning people | Not given | Member checking | Single in-depth interviews | 5 f.  Age range 28 - 44 | All stroke | Range 1 – 9 years | Delayed diagnosis, relationships with health professionals and access to psychosocial rehabilitation, financial hardship |
| 56 | Wood 2010  Canada | Process of community reintegration | Longitudinal grounded theory | No | 46 semi-structured interviews | 10; 6 m, 4 f.  Age range 31 - 79 | All stroke | 5 interviews predischarge, 2 weeks, 3 months, 6 months, 12 months | Hard to accept help in early stages. Transition from feeling overwhelmed to control and independence |

**Abbreviations**: f = female; LOS = length of stay; m = male; Mod = moderate; TBI = traumatic brain injury; rehab = rehabilitation; wks = weeks

**Key**: µ living circumstance not given but presume more than 75% in community; Ϯ same research with different focus and participants only counted once; ≠ mean age/ details given indicates 75% of participants over 18 – all ABI participant data extracted; *time since injury depends on how long in hospital; £ demographic information includes people who were not interviewed; β participants in residential care/ supported accommodation/ hospital not included; ∞ May be the same study with different focus but unable to compare across studies due to different detail given and author did not respond to query - all participants included in total; π number includes interviews with significant others as some were done in dyads ¥ only participants over 18 reported and extracted; ^ data only extracted from people with ABI.
