# Supplementary material for: Perspectives of choice and control in daily life for people following brain injury: A qualitative systematic review and meta‐synthesis
Source: Health Expect. 2022 Oct 31;25(6):2709–25. doi: 10.1111/hex.13636 (PMC9700193; doi:10.1111/hex.13636)
Supplement: Supplementary file 3 — Supporting information. [file HEX-25--s001.docx]

**Supplementary File S3a: Critical appraisal against McMaster criteria A - Kel**

|  | Allen 2021 | Anderson 2013 | Arntzen 2015 | Berg 2017 | Boger 2015 | Burton 2000 | Carulli 2018 | Coneeley 2002 | Coneeley 2003 | Conneeley 2012 | Dumont 2007 | Finch 2020 | Fraas 2009 | Gallagher 2011 | Gould 2019 | Graff 2020 | Green 2009 | Häggström 2008 | Hammond 2021 | Harrington 2015 | Harris-Walker 2020 | Herrmann 2019 | Johansson 2016 | Jones 2008 | Jumisko 2009 | Kamwesiga 2016 | Kelly 2021 |
| --- | --- | --- | --- | --- | --- | --- | --- | --- | --- | --- | --- | --- | --- | --- | --- | --- | --- | --- | --- | --- | --- | --- | --- | --- | --- | --- | --- |
| **STUDY PURPOSE:**  1. Was the purpose and/or research question stated clearly? | √ | √ | √ | √ | √ | √ | √ | √ | √ | √ | √ | √ | √ | √ | √ | √ | √ | √ | √ | √ | √ | √ | √ | √ | √ | √ | √ |
| **LITERATURE:**  2. Was relevant background literature reviewed? | √ | √ | √ | √ | √ | √ | √ | √ | √ | √ | √ | √ | √ | √ | √ | √ | x | √ | √ | √ | √ | √ | √ | √ | √ | √ | √ |
| **STUDY DESIGN:**  3. Was the design appropriate for the study question? (i.e., rationale) | √ | √ | √ | √ | √ | √ | √ | √ | √ | √ | √ | √ | √ | √ | ID | √ | ID | ID | √ | ID | √ | √ | √ | √ | √ | √ | ID |
| 4. Was a theoretical perspective identified? | √ | √ | √ | x | √ | x | x | √ | √ | √ | x | x | √ | √ | x | √ | x | √ | √ | √ | √ | x | √ | x | √ | x | x |
| 5. Are the methods congruent with the purpose? | √ | √ | √ | √ | √ | √ | √ | √ | √ | √ | √ | x | √ | √ | √ | √ | √ | √ | √ | √ | √ | √ | √ | √ | √ | √ | √ |
| **SAMPLING:**  6. Was the process of purposeful sampling described? | √ | √ | √ | √ | √ | ID | ID | √ | √ | √ | √ | ID | x | √ | √ | √ | x | √ | ID | √ | x | ID | √ | √ | ID | ID | x |
| 7. Was sampling done until redundancy in data was reached? | x^ | √ | x | √ | √ | x | x | x | x | x | x ^ | x | x | x | ID | ID | x | x | x | √ | √ | ID | x | x | x | √ | x |
| 8. Was informed consent and ethical approval obtained? | √ | √ | √# | √ | √ | √ | √# | √ | √ | √ | √ | √ | √ | √ | √ | √ | √ | √ | √ | √ | √ | √ | √ | √ | √ | √ | √ |
| **DATA COLLECTION:**  **Descriptive Clarity**  9. Clear & complete description of site? | x | √ | x | √ | √ | x | ID | √ | √ | √ | x | ID | ID | x | ID | √ | x | x | x | x | ID | √ | √ | x | x | √ | √ |
| 10. Participants? (demographics) | √ | √ | √ | √ | √ | ID | ID | √ | √ | √ | √ | √ | √ | ID | √ | √ | √ | √ | √ | √ | x | ID | √ | √ | √ | √ | √ |
| 11. Role of researcher & relationship with participants? | √ | x | x | √ | x | x | x | √ | √ | √ | x | √ | ID | x | x | √ | x | x | x | x | √ | x | x | x | x | √ | √ |
| 12. Identification of assumptions and biases of researcher | √ | x | x | x | x | x | ID | √ | √ | √ | x | x | x | x | x | x | x | x | x | x | x | x | x | x | ID | x | ID |
| **Procedural Rigour**  13. In data collection? | √ | ID | x | ID | √ | ID | x | ID | ID | ID | ID | ID | √ | x | √ | √ | ID | x | √ | ID | ID | ID | √ | ID | ID | √ | √ |
| **DATA ANALYSES:**  **Analytical Rigour**  14. Data analyses were inductive? | √ | √ | √ | √ | √ | √ | √ | √ | √ | √ | √ | √ | √ | √ | √ | √ | √ | √ | √ | √ | √ | ID | √ | √ | √ | √ | √ |
| 15. Findings were consistent with & reflective of data? | √ | √ | √ | √ | √ | √ | √ | √ | √ | √ | √ | √ | √ | √ | √ | √ | √ | √ | √ | √ | √ | ID | √ | √ | ID | √ | √ |
| **Auditability**  16. Decision trail developed? | x | ID | ID | √ | ID | ID | ID | ID | ID | ID | x | √ | ID | √ | √ | x | ID | ID | x | ID | √ | x | √ | √ | ID | √ | ID |
| 17. Process of analysing the data was described adequately? | √ | √ | √ | √ | √ | ID | ID | x | ID | ID | √ | √ | √ | √ | √ | √ | √ | √ | ID | √ | √ | ID | √ | √ | √ | √ | √ |
| **Theoretical Connections**  18. Did a meaningful picture of the phenomenon under study emerge? | √ | √ | √ | √ | √ | x | √ | √ | √ | √ | √ | √ | ID | √ | √ | √ | √ | √ | √ | √ | √ | √ | √ | √ | √ | √ | √ |
| **OVERALL RIGOUR**  19. Was there evidence of the four components of trustworthiness?  -Credibility | √ | x | ID | √ | √ | ID | √ | √ | √ | √ | ID | ID | x | √ | √ | ID | ID | ID | √ | ID | √ | √ | √ | √ | ID | √ | √ |
| -Transferability | √ | √ | ID | √ | √ | x | ID | √ | √ | √ | √ |  | √ | ID | ID | √ | ID | ID | ID | ID | √ | ID | √ | ID | ID | √ | √ |
| -Dependability | √ | √ | √ | √ | ID | x | ID | x | x | x | x | √ | √ | √ | ID | √ | ID | √ | √ | ID | √ | ID | √ | √ | x | √ | √ |
| -Confirmability | ID | x | ID | ID | ID | ID | √ | ID | ID | ID | ID | ID | ID | ID | √ | √ | ID | √ | √ | √ | √ | x | √ | √ | √ | √ | √ |
| **CONCLUSIONS & IMPLICATIONS**  20. Conclusions were appropriate given the study findings? | √ | √ | √ | √ | √ | √ | √ | √ | √ | √ | √ | √ | √ | √ | √ | √ | √ | √ | √ | √ | √ | √ | √ | √ | √ | √ | √ |

**Key:** ✓ = Sufficient detail provided; X = Question not met; ID = Insufficient detail; *ethics supplied by author; ^reasons given; #consent not addressed

**Table 1b: Critical appraisal against McMaster criteria Kes - Z**

|  | Kessler 2009 | King 2018 | Kitson 2013 | Knox 2016 | Knox 2017 | Koller 2016 | Kubina 2013 | Kusec 2020 | Lawson 2008 | McCluskey 2007 | Mealings 2021 | Moss 2021 | Nalder 2013 | Olofsson 2005 | Paniccia 2019 | Pereira 2020 | Price 2012 | Quinn 2015 | Ringsberg 2003 | Satink 2016 | Sveen 2016 | Taule 2015 | Timothy 2016 | Tomkins 2013 | Turner 2009 | Vestling 2013 | Walder 2017 | Wolfenden 2015 | Wood 2010 |
| --- | --- | --- | --- | --- | --- | --- | --- | --- | --- | --- | --- | --- | --- | --- | --- | --- | --- | --- | --- | --- | --- | --- | --- | --- | --- | --- | --- | --- | --- |
| **STUDY PURPOSE:**  1. Was the purpose and/or research question stated clearly? | √ | √ | √ | √ | √ | √ | √ | √ | √ | √ | √ | √ | √ | √ | √ | √ | √ | √ | √ | √ | √ | √ | √ | √ | √ | √ | ID | √ | √ |
| **LITERATURE:**  2. Was relevant background literature reviewed? | √ | √ | √ | √ | √ | √ | √ | √ | √ | √ | √ | √ | √ | x | √ | √ | √ | √ | √ | √ | √ | √ | √ | √ | √ | √ | √ | √ | √ |
| **STUDY DESIGN:**  3. Was the design appropriate for the study question? (i.e., rationale) | √ | √ | √ | √ | √ | √ | √ | √ | √ | √ | √ | √ | ID | ID | √ | ID | ID | √ | √ | x | x | √ | √ | √ | √ | x | √ | ID | √ |
| 4. Was a theoretical perspective identified? | √ | √ | √ | √ | √ | √ | x | √ | √ | x | √ | x | √ | x | x | √ | √ | √ | x | √ | √ | √ | √ | x | x | x | √ | √ | √ |
| 5. Are the methods congruent with the purpose? | √ | √ | √ | √ | √ | √ | √ | √ | √ | √ | √ | √ | √ | √ | √ | √ | √ | √ | x | √ | √ | √ | √ | √ | √ | √ | √ | √ | √ |
| **SAMPLING:**  6. Was the process of purposeful sampling described? | x | √ | √ | √ | ID | ID | √ | ID | N/A | √ | √ | √ | √ | √ | √ | √ | x | ID | ID | √ | x | √ | √ | √ | x | √ | √ | x | √ |
| 7. Was sampling done until redundancy in data was reached? | x | x | ID | x | ID | x | √ | √ | N/A | x^ | √ | ID | x | √ | √ | x | x | x | x | x | x | √ | √ | √ | √ | x | √ | x | √ |
| 8. Was informed consent and ethical approval obtained? | √* | √ | √ | √ | √ | √ | √# | √ | √ | √ | √ | √ | √ | √ | √ | √ | √ | √# | √ | √ | √ | √ | √ | √* | √ | √ | √ | √# | √ |
| **DATA COLLECTION:**  **Descriptive Clarity**  9. Clear & complete description of site? | x | x | x | ID | x | √ | x | √ | √ | √ | √ | √ | x | x | x | √ | x | ID | √ | ID | ID | √ | √ | x | x | x | x | √ | x |
| 10. Participants? (demographics) | √ | √ | x | √ | √ | √ | √ | √ | √ | √ | √ | √ | √ | √ | √ | √ | √ | √ | √ | √ | √ | √ | √ | x | √ | √ | √ | √ | √ |
| 11. Role of researcher & relationship with participants? | x | ID | x | √ | √ | x | x | √ | √ | x | x | √ | x | x | x | ID | x | x | √ | ID | x | √ | √ | x | x | x | x | √ | √ |
| 12. Identification of assumptions and biases of researcher | x | x | x | x | x | x | x | ID | ID | x | x | √ | x | x | ID | √ | x | √ | x | x | x | √ | x | x | x | x | x | √ | ID |
| **Procedural Rigour**  13. In data collection? | ID | ID | √ | √ | √ | ID | ID | ID | ID | √ | √ | √ | x | x | √ | √ | x | ID | x | √ | x | ID | √ | √ | √ | ID | x | ID | √ |
| **DATA ANALYSES:**  **Analytical Rigour**  14. Data analyses were inductive? | √ | √ | √ | √ | √ | √ | √ | √ | √ | √ | √ | √ | √ | √ | √ | √ | √ | √ | ID | √ | √ | √ | √ | √ | √ | √ | √ | √ | √ |
| 15. Findings were consistent with & reflective of data? | √ | √ | √ | √ | √ | √ | √ | √ | √ | √ | √ | √ | √ | √ | √ | √ | ID | √ | ID | √ | √ | √ | √ | √ | √ | √ | √ | √ | √ |
| **Auditability**  16. Decision trail developed? | ID | √ | x | ID | x | √ | √ | x | ID | x | x | √ | √ | x | √ | x | x | ID | x | √ | x | x | x | √ | √ | x | √ | x | x |
| 17. Process of analysing the data was described adequately? | √ | √ | √ | √ | √ | √ | √ | √ | √ | √ | √ | √ | √ | √ | √ | √ | ID | √ | x | √ | √ | √ | √ | ID | √ | √ | √ | √ | √ |
| **Theoretical Connections**  18. Did a meaningful picture of the phenomenon under study emerge? | √ | √ | √ | √ | √ | √ | √ | √ | √ | √ | √ | √ | √ | √ | √ | √ | x | √ | x | √ | √ | √ | √ | √ | √ | √ | √ | √ | √ |
| **OVERALL RIGOUR**  19. Was there evidence of the four components of trustworthiness?  -Credibility | √ | √ | ID | √ | √ | √ | √ | √ | √ | √ | √ | √ | √ | ID | √ | √ | ID | √ | x | √ | ID | √ | √ | √ | √ | √ | √ | √ | √ |
| -Transferability | x | ID | x | ID | √ | ID | ID | √ | √ | √ | √ | √ | ID | ID | √ | √ | x | √ | ID | √ | ID | √ | x | ID | √ | √ | x | √ | √ |
| -Dependability | ID | √ | √ | ID | √ | √ | √ | ID | √ | √ | x | √ | √ | ID | √ | √ | x | √ | ID | √ | √ | √ | √ | √ | √ | ID | √ | √ | √ |
| -Confirmability | ID | √ | √ | √ | √ | √ | ID | √ | √ | ID | √ | √ | √ | x | √ | √ | ID | √ | x | √ | √ | √ | √ | √ | ID | √ | √ | √ | √ |
| **CONCLUSIONS & IMPLICATIONS**  20. Conclusions were appropriate given the study findings? | √ | √ | √ | √ | √ | √ | √ | √ | √ | √ | √ | √ | √ | √ | √ | √ | √ | √ | √ | √ | √ | √ | √ | √ | √ | √ | √ | √ | √ |

**Key:** ✓ = Sufficient detail provided; X = question not met; ID = Insufficient detail; *ethics supplied by author; N/A = not applicable; ^reasons given; # consent not addressed
